# Methylation in *HOX* Clusters and Its Applications in Cancer Therapy

**DOI:** 10.3390/cells9071613

**Published:** 2020-07-03

**Authors:** Ana Paço, Simone Aparecida de Bessa Garcia, Renata Freitas

**Affiliations:** 1 Centre Bio: Bioindustries, Biorefineries and Bioproducts, BLC3 Association—Technology and Innovation Campus, 3405-169 Oliveira do Hospital, Portugal; anaisapaco@gmail.com; 2 I3S—Institute for Innovation & Health Research, University of Porto, 4200-135 Porto, Portugal; simone.bessa@i3s.up.pt; 3 ICBAS—Institute of Biomedical Sciences Abel Salazar, University of Porto, 4050-313 Porto, Portugal

**Keywords:** *HOX* genes, DNA methylation, histone methylation, epigenetics, cancer

## Abstract

*HOX* genes are commonly known for their role in embryonic development, defining the positional identity of most structures along the anterior–posterior axis. In postembryonic life, *HOX* gene aberrant expression can affect several processes involved in tumorigenesis such as proliferation, apoptosis, migration and invasion. Epigenetic modifications are implicated in gene expression deregulation, and it is accepted that methylation events affecting *HOX* gene expression play crucial roles in tumorigenesis. In fact, specific methylation profiles in the *HOX* gene sequence or in *HOX*-associated histones are recognized as potential biomarkers in several cancers, helping in the prediction of disease outcomes and adding information for decisions regarding the patient’s treatment. The methylation of some *HOX* genes can be associated with chemotherapy resistance, and its identification may suggest the use of other treatment options. The use of epigenetic drugs affecting generalized or specific DNA methylation profiles, an approach that now deserves much attention, seems likely to be a promising weapon in cancer therapy in the near future. In this review, we summarize these topics, focusing particularly on how the regulation of epigenetic processes may be used in cancer therapy.

## 1. Introduction

The development of cancer is tightly linked to an accumulation of changes in the structure and function of the genome that result in transcriptional regulation errors and altered gene expression [1]. In addition, these genomic alterations can lead to epigenetic modifications, which modify DNA accessibility and further change the chromatin structure, thereby contributing to aberrant gene expression. In the first stage of cancer research, great attention was paid to the description of mutations in oncogenes and tumor suppressor genes, and also to the functional characterization of genes and proteins. However, more recently, epigenetic modifications have emerged as a crucial mechanism for cancer onset, progression and metastasization [2,3]. These modifications are reversible and do not affect the DNA sequence, but are vital for genomic structure maintenance and gene expression control, being heritable through successive cell divisions [4].

Four main epigenetic events have been linked to gene expression alterations: DNA methylation, posttranslational modifications of histones, chromatin remodeling and RNA-based mechanisms [5]. DNA methylation is promoted by DNA methyltransferases (DNMTs 1, 2, 3 and their variants), which add methyl groups (CH_3_) to the cytosine residues at Carbon 5, yielding 5′ methyl-cytosines. Briefly, DNMT1 is involved in methylation maintenance after DNA replication, DNMT2 is a tRNA methyltransferase and DNMT3 relates to *de novo* DNA methylation [6]. In vertebrate genomes, the addition of methyl groups mostly occurs on cytosine residues that precede guanine, known as CpG dinucleotides. These CpG sites can be clustered in specific regions of the genome, as short interspersed DNA sequences, known as CpG islands, with an average of 1000 base pairs (bps). Gene promoter regions frequently have CpG islands in which gene expression regulation can occur by methylation. [7].

**Figure 1:**
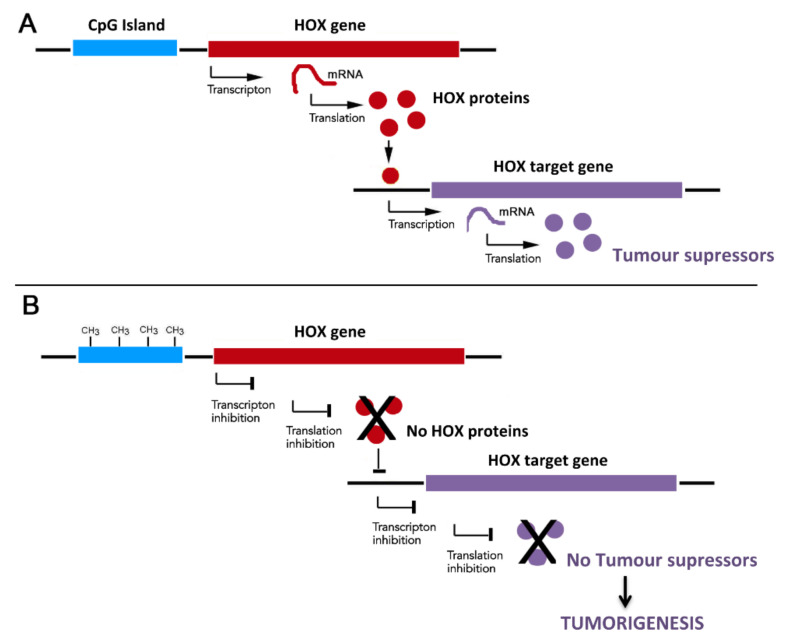
Possible consequences of *HOX* expression inhibition by CpG island DNA methylation. (**A**) Expression activation of *HOX* target genes with roles in tumorigenesis. When the CpG islands are demethylated, the chromatin is accessible to transcription factors and other proteins related to transcriptional activation with the consequent translation of genes that can be tumor suppressors or pro-apoptotic. (**B**) Expression inhibition of *HOX* target genes with roles in tumorigenesis. When the CpG islands are methylated, the chromatin becomes inaccessible for transcription activators in such a way that tumor suppressors and apoptotic genes cannot be transcribed and translated. CH_3_ - Methyl groups.

DNA methylation, leading to gene promoter hypermethylation and consequent transcriptional inhibition, has been observed in a wide variety of cancers with impact on their progression and aggressiveness (Figure 1) [8]. The genetic silencing mediated by DNA methylation occurs in combination with other epigenetic events, such as histone modifications and chromatin remodeling that gives rise to tight chromatin structures, hampering transcriptional activity [2].

**Figure 2:**
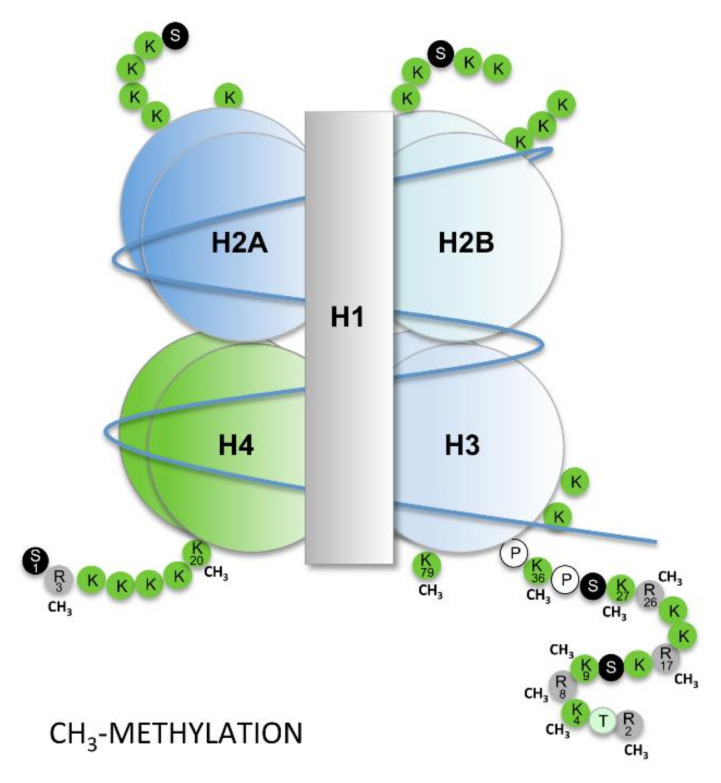
Nucleosome organization. Each nucleosome is composed of an octamer comprising four histones proteins, i.e., H2A, H2B, H3 and H4, wrapped twice by the DNA molecule. Methyl groups (CH_3_) can be added or removed from the lysine (K) and arginine (R) residues of histone H3 and H4 in a nucleosome. Histone modifications, including methylation and acetylation, are important mechanisms for gene transcription regulation independent of the promoter methylation status. P, proline; S, serine; T, threonine. DNA molecule represented in red.

Histone modifications also affect the assembly and restructuration of the nucleosome [9,10]. This fundamental repeat unit of the chromatin corresponds to an octamer of four core histone proteins (H2A, H2B, H3 and H4) wrapped twice around the DNA molecule (Figure 2) [11]. The histones may acquire modifications, namely by the acetylation and methylation of lysines (K) and arginines (R), as well as by the phosphorylation of serines (S) and threonines (T) [9]. Other modification may include ubiquitylation, via an isopeptide bond to lysine residues (K), and sumoylation, involving the addition of SUMOs (small ubiquitin-like modifiers). A wide variety of enzymes participate in these processes such as acetyltransferases, deacetylases, methyltransferases, demethylases and kinases. All these enzymes work in concert with ATP-dependent chromatin-remodeling complexes that recognize specific histone modifications, affecting the disassembly and assembly of nucleosomes and the movement of histone octamers along the DNA [10].

Regarding the methylation pattern of histones, some methylations are features of active chromatin, such as the trimethylation of lysine 4 or 36 on histone H3 (H3K4me3 and H3K36me3), together with the hyperacetylation of histones H3 and H4 (H3ac, H4ac), while others are features of a silencing chromatin state, such as the trimethylation of lysines 9 and 27 on histone H3 (H3K9me3 and H3K27me3), together with the hypoacetylation of this histone [11]. The H3K27me3 is promoted by a complex of proteins, i.e., the PRC2 complex (polycomb repressive complex 2) [12], which plays a particular role in *HOX* gene expression regulation, as will be described later.

Other epigenetic events interfering with gene expression involve the interaction of noncoding RNAs with the chromatin, forming complexes able to regulate transcription, designated as RNA-based mechanisms [5]. These RNAs can be antisense mRNAs that binds to the sense transcripts impairing their translation to protein. They can also be microRNAs that act as posttranscriptional regulators inducing mRNA degradation and/or translational repression [13].

In summary, epigenetic mechanisms affect gene expression by interfering with its regulation pre- or post- transcriptionally. In addition, they can affect the disassembly/assembly of nucleosomes and their movement/interaction with DNA. Chromatin remodeling may increase the accessibility of DNA, facilitating interaction among transcription factors or, in contrast, promoting the packing of chromatin into tightly folded structures, thereby hampering interactions [14]. Therefore, non-methylated CpG island promoters present looser chromatin, whereas methylated promoters have a more packed chromatin [2].

**Figure 3:**
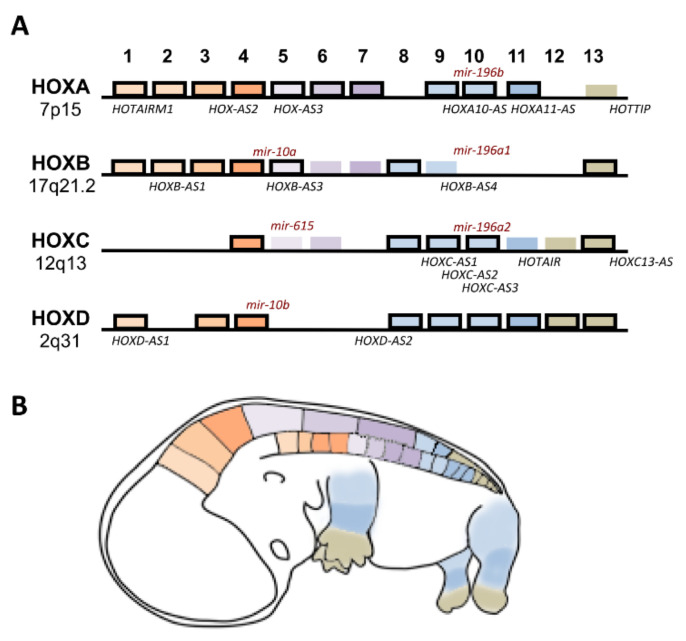
Human *HOX* clusters and their expression during development. (**A**) *HOX* genes organized in four clusters (A, B, C, D). Cluster designation is on the left, and Paralogous (1–13) are indicated at the top. *HOX* genes, which were proven to be methylated or demethylated in cancer, are outlined in black, and the noncoding RNAs involved in cancer are marked in the approximate position from which there are transcribed (mir, microRNAs; AS, antisense RNAs). (**B**) *HOX* gene expression along the anterior–posterior axis (head and trunk) and along the proximal–distal axis of limbs.

*HOX* gene transcriptional regulation involves not only *cis* and *trans* regulatory elements, but also several epigenetic mechanisms (Figure 3). For some *HOX* genes, and considering particular contexts such as embryonic development or cancer, epigenetic variations and their downstream effects are still under investigation. The human genome contains 39 genes organized into four clusters (*HOXA*, *HOXB*, *HOXC* and *HOXD*) located within distinct chromosomes (7p15, 17q21.2, 12q13, 2q31, respectively), encoding transcription factors and noncoding RNAs that are crucial for embryonic development, cellular physiology and tissue homeostasis (Figure 3) [15,16].

A large number of studies, including genome-wide association approaches, have highlighted connections between *HOX* gene expression and cancer, either being downregulated or upregulated in comparison with its normal counterparts, where they may act as tumor suppressors or proto-oncogenes in a tissue-specific context [17]. These alterations in *HOX* gene expression could be the result of epigenetic processes that affect chromatin accessibility, or genetic processes that affect the *HOX* gene DNA sequence, cofactor assembly and upstream regulators. Changes in the expression profile of these genes and functional abnormalities in the encoded transcription factors have been shown to affect several cellular processes, such as angiogenesis, autophagy, proliferation, apoptosis, migration and metabolism [15,17,18]. *HOX* gene mutations have been investigated in the past decade and found to increase cancer susceptibility, beyond being related to limb malformations, among other physiologic disorders [19,20].

Interestingly, DNA methylation appears to be an important mechanism for *HOX* gene regulation, with a particular impact on cancer progression. Therefore, the methylation status of a wide range of *HOX* genes is assuming increasing importance as a potential cancer prognostic marker [17]. In this review, we describe the state of knowledge of *HOX* gene methylation in cancer, clearly illustrating the remarkable potential of these epigenetic events for cancer prognostic marker discovery. We also report the histone methylation processes shown to have an impact on *HOX* gene transcription associated with cancer, and we discuss the therapies targeting methylation in HOX-associated cancers.

## 2. *HOX* Genes Methylation in Cancer

Cancer is recognized as being not only genetically but also epigenetically distinct from its tissue of origin [21]. However, while the genetic alterations underlying oncogene upregulation have been heavily studied, the epigenetic mechanisms that can also induce oncogene expression remain largely unknown [21]. The methylation of gene promoters is one of the epigenetic mechanisms most frequently identified during the progression of human cancers. However, this mechanism of *HOX* gene regulation is not exclusively related to cancer development. Differential *HOX* gene methylation was also identified in neurofibroma, commonly a benign disease [22], and in endometrium with lower receptivity for embryo implantation [23].

The methylation profile of most *HOX* genes has been investigated in a variety of cancer types, and is considered a valuable biomarker for their diagnosis and prognosis (Table 1) [22,24,25,26,27]. The *HOX* gene hypermethylation is often linked to the silencing of *HOX* gene targets working as tumor-suppressor and/or apoptotic genes (Figure 1) [3,28]. Interestingly, a recent study of DNA methylation profiles across the genome in normal and tumor tissues suggests an unexpected causal role of gene hypermethylation for *HOX* oncogene activation [21]. Moreover, situations occur in which *HOX* genes are found to be hypomethylated during tumorigenesis; this is the case of *HOXC10* in gastric cancer [29,30]. In this case, hypomethylation leads to *HOXC10* overexpression, the downstream effects of which include increased of proliferation and the migration of cancer cells.

### 2.1. HOXA Genes Methylated in Cancer

Genes from the *HOXA* cluster have a tendency for hypermethylation, and consequent downregulation, in most cancer types studied. It has been proposed that the methylation state of *HOXA1*, in combination with other genes, is a useful marker in the detection of malignant biliary obstruction, increasing the sensitivity of diagnoses by cytology [31] and in the diagnosis of thyroid nodules [101]. In addition, the *HOXA1* methylation profile was also considered important in the identification of specific states of cancer progression in lung and breast carcinomas [40,69], also in combination with the hypermethylation of other *HOX* genes such as *HOXA10* and *HOXB13* [24]. In gastric cancer, the aberrant *HOXA1* methylation profile is associated with clinicopathological characteristics and clinical outcomes [95].

Similarly, *HOXA2* was found to exhibit distinct methylation profiles in at least four cancer sites: lung, colon/rectum, nasopharynx and bile duct [32,55,62,70,71]. In lung squamous cell carcinoma (SCC), *HOXA2* was included in a panel of hypermethylated genes that might be useful to stratify SCC into molecular subtypes with distinct prognoses [70]. It has been suggested that the *HOXA2* methylation status, together with the methylation profile of other *HOXA* genes, may have prognostic value in laryngeal squamous cell carcinoma [105]. In addition, *HOXA2* methylation analyses may work as a differential epigenetic biomarker between malignant and nonmalignant biliary and nasopharyngeal tissues [32,62]. In colorectal cancer, the study of the promoter methylation patterns of *HOXA2*, *A5* and *A6* were considered important in assessments of risk for this malignancy [55].

For *HOXA3*, differential methylation profiles were found in glioma, lung and penile carcinomas, leading to its consideration as part of the methylome signature associated with these diseases [72,80,84,106]. In gliomas, *HOXA3*, *A7*, *A9*, and *A10* are methylation targets mainly in high-grade tumors, and their role as potential biomarkers has been proposed to clinically distinguish among patient subgroups [84]. In breast cancer, the increased DNA methylation of *HOXA4* was proposed as a biomarker for early disease detection [41], and *HOXA5* hypermethylation was identified specifically as part of the molecular portrait associated with high-grade ductal carcinoma in situ [42] and Triple-negative breast cancer patients nonresponsive to neoadjuvant chemotherapy [43]. In addition, the hypermethylation of *HOXA5*, together with five other genes (*ABCA3*, *COX7A1*, *SLIT3*, *SOX17*, *SPARCL1*), has been linked to lung adenocarcinoma development [107], while *HOXA4* and *HOXA5* present altered methylation profiles in a significant number of patients with acute myeloid leukemia [108].

The potential of *HOX* gene methylation profiles was also explored for the early detection of lung cancer in plasma and sputum, as *HOXA7* and *HOXA9* hypermethylation are part of the signature associated with this disease [109]. The aberrant methylation of *HOXA9* is characteristic of a wide variety of cancers, and is used as a biomarker for diagnoses and prognoses in distinct sample types. In serum, for example, the hypermethylation of *HOXA9* was recently proposed as a marker to detect early epithelial ovarian cancer [27]. Moreover, the methylation profile of this gene was considered, in combination with other genes, to be potentially applicable for prostate cancer clinical staging based on urine collection [91]. The methylation profile of *HOXA9* has also been proposed as a reliable biomarker to identify resistance to cisplatin-based therapy in bladder cancer [34], and as a tool for testicular germ cell tumors subtyping [100]. In addition, the methylation status of *HOXA9* was considered relevant for subtyping lung cancer using liquid biopsies [110] or for its early detection in circulating cell-free DNA [58]. *HOXA9* hypermethylation was also found to be a tool to identify advanced neck squamous cell carcinomas favoring tumor progression and metastasis [57], predict survival in breast cancer patients, together with *HOXA10* hypermethylation [111], and detect early onset of endometrial cancer [58].

Knowledge of the downstream processes affected by *HOXA* gene deregulation, due to alterations in their methylation profile, is still incomplete for most cancers. However, for *HOXA10*, for example, promoter hypermethylation favors miR-196b-5p–dependent cell proliferation and invasion in gastric cancer cells [30]. In addition, in lung adenocarcinoma, *HOXA11* hypermethylation seems to be related to cisplatin-resistance and to *Akt*/*β-catenin* signaling activation, which occurs without interfering with the methylation status of *HOXA11* antisense (*HOXA11AS)* [112].

### 2.2. HOXB and HOXC Genes Methylated in Cancer

Genes from the *HOXB* cluster (*HOXB2*, *B3*, *B4*, *B9*, *B13*) have been found to be hypermethylated in a variety of tumors. The hypermethylation of *HOXB2* was considered part of a signature exclusively found in the lymph node metastasis of the esophageal squamous cell carcinoma, serving as a possible biomarker for early diagnoses and prognoses [63]. In addition, it is also one of the methylated genes associated with bladder cancer aggressiveness [36]. *HOXB3* and *HOXB4* hypermethylation were identified as potential biomarkers in lung adenocarcinoma diagnosis [80]. In addition, data from nearly 63,000 women of European ancestry suggest that *HOXB3* hypermethylation is among the epigenetic modifications associated with epithelial ovarian cancer risk [113], and that *HOXB4* is part of a multigene methylation signature found in circulating tumor cells from patients with metastatic breast cancer [46]. Among *HOXB* genes, *HOXB13* is frequently identified as being hypermethylated in tumors. Its promoter methylation is a candidate biomarker for gastric [64] and endometrial tumors with enhanced invasiveness [114]. The hypermethylation of *HOXB13* also occurs in nearly 30% of renal cell carcinomas, as the silencing of this gene is associated with apoptosis ratio decrease, tumor grade increase and microvessels invasion [61].

Most genes from the *HOXC* cluster have been identified as hypermethylated in cancer (*HOXC4*, C5, C6, C8, C9) [48,115,116,117]. Methylated regions in a gene collection that includes *HOXC4* were considered important in estimating cancer risk in urothelium [117] and as part of a prognostic signature predicting survival in patients with oral squamous cell carcinoma [66]. The role of *HOXC8* in breast cancer, in which silencing seems to interfere with the self-renewal, differentiation and transformation of breast cancer stem cells, is also instigated by promoter hypermethylation [48]. However, there are also *HOXC* genes that are hypomethylated in cancer; this is the case of *HOXC10* in gastric cancer, which is associated with cell proliferation and migration [29].

### 2.3. HOXD Genes Methylated in Cancer

*HOXD* genes are also regularly found to be hypermethylated in a wide variety of cancers, and have been widely proposed as valuable biomarkers for the prognosis and diagnosis of this disease. *HOXD1* hypermethylation is part of a signature helping to predict lymph node metastasis in gastric cancer [29]. Similarly, *HOXD3* hypermethylation is part of a panel that includes *HOXD8* methylation [94], which makes it possible to test the clinical significance of prostate cancer using urine samples [29], and is also considered to be among the prognostic indicators of late recurrence or of the need for hormone therapy after surgery in prostate cancer biopsies [118]. Interestingly, the hypermethylation of *HOXD3* is a feature of the most common male cancers worldwide (lung, prostate and colorectal cancers) [56], but also a prognostic marker in renal cell [56] and hepatocellular carcinomas [119]. Moreover, *HOXD9* hypermethylation is a common epigenetic alteration in thymic carcinoma [17] and one of the biomarkers that may help to differentiate cholangiocarcinoma from other biliary diseases using serum cell-free DNA analysis [33].

The DNA methylation level of *HOXD10* is part of a profile that is significantly correlated with a higher aggressiveness of early-onset endometrial cancer [58]. In addition, it is a recognizable marker in papillary thyroid cancer patients, particularly among BRAFV600E mutation carriers [104]. It has also been suggested that *HOXD10* hypermethylation detection in the plasma, in combination with other genes, may be a useful biomarker for the early detection of gastric cancer and pre-cancerous lesions [58], and to distinguish lung cancer, pulmonary fibrosis and chronic obstructive lung disease [82]. The downstream impact of these epigenetic aberrations is still not fully characterized. However, epigenetic inactivation of *HOXD10* has been associated with colon cancer, inhibiting RHOC/AKT/MAPK signaling [57], and with hepatocellular carcinoma, activating ERK signaling [67]. *HOXD13* hypermethylation has been particularly associated with breast cancer, as part of an epigenetic signature detectable in the serum and used for clinical diagnoses [120], and in lung adenocarcinoma, in which it is considered a potential prognostic biomarker [83].

## 3. Histone Methylation with Impact on HOX Gene Transcription in Cancer

**Figure 4:**
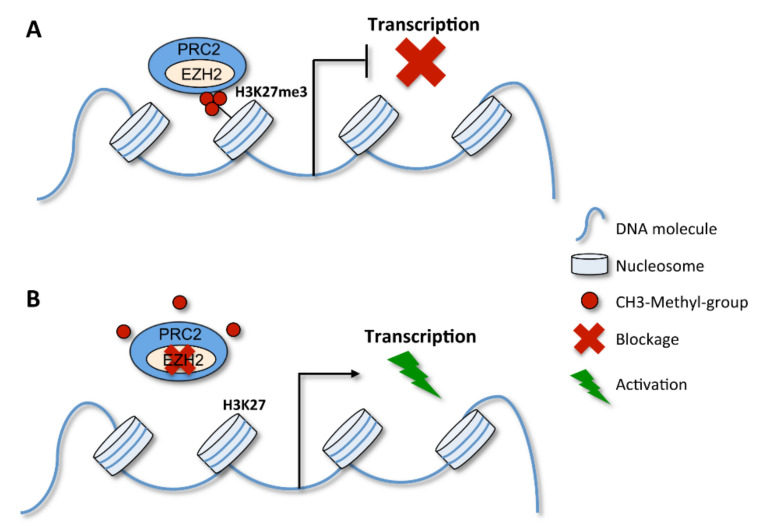
PRC2 complex action in gene expression regulation. EZH2 is a catalytic subunit of the PRC2 complex and fundamental for the methyltransfer process. (**A**) The PRC2 complex can promote the trimethylation of H3K27 (H3K27met3), which impairs gene transcription. (**B**) When the PRC2 complex is inhibited, for example by the EZH2 blockage, H3K27 becomes demethylated and gene expression proceeds.

As mentioned, *HOX* genes do not only play a role in cancer when downregulated or silenced. In particular contexts, their upregulation is tightly linked with cancer progression [15]. This can easily occur by an alteration of the methylation pattern of their associated histones. As previously mentioned, the PRC2 protein complex plays a particular role in *HOX* gene regulation. Due to its histone methyltransferase activity, it is able to methylate histone H3 on lysine 27 (H3K27me3) that interferes with *HOX* gene expression, with an impact on cancer predisposition and progression (Figure 4) [121]. Interestingly, some *HOX* transcripts have the unusual ability to control the expression of other *HOX* genes by recruiting the PRC2 complex. As an example, the transcription of the antisense strand located between *HOXC11* and *HOXC12* on human chromosome 12 gives rise to long noncoding RNAs, named HOTAIRs, which trigger the silencing of *HOXD* genes by recruiting the PRC2 complex [122].

In acute myeloid leukemia, patients carrying a mutation in the sex combs-like 1 gene (*ASXL1*) often have genome-wide loss of H3K27me3, including in the *HOXA* cluster region. ASXL1 physically interacts with the Enhancer of Zeste Homolog 2 (EZH2), a histone-lysine N-methyltransferase enzyme and a core member of the PRC2 complex, causing H3K27me3 loss. This results in an increase of *HOXA9* and *HOXA10* expression [17], favoring leukemia progression due to increased cell proliferation [123,124]. A similar mechanism was suggested for non-small cell lung cancer in which *HOXB7* promoter was found to interact with *EZH2* and have its H3K27 trimethylated. The specific modulation of *HOXB7* interferes with the AKT and MAPK pathways, impacting tumor growth [125]. Moreover, H3K4me3 and H3K36me3 of the promoter regions of the *HOXB7*, *HOXC10* and *HOXD8* genes are also considered potential biomarkers in oral squamous cell carcinoma. These histone methylations favor *HOX* gene expression, which has been associated with the neoplastic phenotype of oral keratinocytes [126]. In contrast, histone methylation can also be associated with *HOX* gene silencing in cancer. In breast cancer cells, for example, H3K27me3 is involved in miR-10a-induced *HOXD4* silencing [127].

## 4. Therapies Targeting Methylation in *HOX*-Associated Cancers

**Figure 5:**
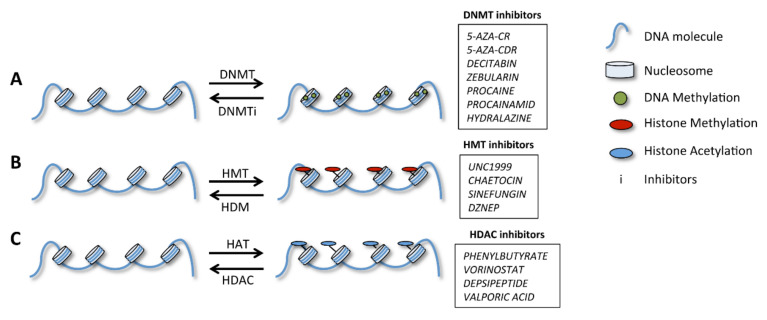
Impact of epigenetic changes in gene expression and related therapeutic agents. (**A**) DNA methylation promoted by DNA-methyltransferases (DNMT) accompanies transcriptional inhibition. (**B**) Histone methylation is promoted by histone methyltransferases (HMT) and leads to chromatin condensation than can either repress or activate transcription. This process is reversed by histone demethylases (HMD). (**C**) Histone acetylation, promoted by histone acetyltransferases (HAT) enzymes, is associated with the opening of the chromatin mass and the onset of transcription, while deacetylation, promoted by histone deacetylases (HDAC), does the opposite.

Besides cancer, many other human diseases are associated with altered DNA or histone methylations. Therefore, an increasing number of studies are now attempting to identify drugs to reverse these alterations. Several studies and clinical trials are undertaking drug testing to modulate the epigenetic profiles in distinct contexts, namely, by interfering with deacetylase proteins and DNA methylation [11]. Thus, a new epigenetic field is currently emerging, i.e., pharmacoepigenomics, which aims to develop and test drugs specifically targeting epigenetic alterations related to cancer [128]. The drugs developed so far are inhibitors of DNA-methyltransferases (DNMTs), histone methyltransferases (HMTs), demethylases (HDMs) or deacetylases (HDACs); these drugs act upon crucial molecules for epigenetic modifications, as previously described (Figure 5) [128,129].

These drugs [11] are potentially useful to reverse the epigenetic status of gene promoters, or in their associated histones. In fact, anti-epigenetic drugs, such as zebularine (in pre-clinical study) and 5-aza-deoxycytidine (approved in 2004) alter the pan-DNA methylation status in cancer [130]. In acute myeloid leukemia, a specific drug (GSK-J4) targeting key histone modulators (KDM6B, the demethylase of H3K27me3) attenuated the disease progression concomitantly with the silencing of cancer-promoting *HOX* genes [131]. In addition, *HOX* antisense intergenic RNA (HOTAIR) can also be targeted in breast cancer models using the compound AC1NOD4Q [132]. This specifically impairs HOTAIR/EZH2 interaction, thereby inhibiting the H3K27-mediated trimethylation of NLK, the target of HOTAIR, and consequently diminishing tumor metastasis through the Wnt/β-catenin pathway. Also, in glioblastoma multiforme, the BET inhibitor, JQ1, impairs HOTAIR, which functions as an epigenetic modulator and contributes to aggressiveness and chemo-resistance [133].

Regarding the alteration in the methylation status of *HOX-*associated histones, it is important to mentioned two human JmjC-domain-containing proteins, UTX and JMJD3. These proteins are essential for healthy development, affecting the epigenetic profiles of *HOX* genes, via H3K27me3 [134]. Moreover, UTX can also be associated with the MLL2 methyltransferase in the H3K4me3 process [135]. The action of these proteins allows the replacement of epigenetic repressive markers to occur by activating markers on *HOX-*associated histones, which seems to be crucial for embryonic development. Therefore, these proteins appear to be important drug targets for the epigenetic control of *HOX* genes [136]. Kruidenier and colleagues [137] are already designing chemical compounds (GSK-J1 and GSK-J3) that inhibit JMJD3 demethylase activity. One of these compounds (GSK-J1) can also inhibit UTX demethylase activity. In addition, animal experiments using these chemical compounds have already been performed, showing positive effects in the inhibition of tumorigenesis. One example is the work of Zhang and colleagues [138], showing that the GSK-J1, in association with TCP (a LSD1 inhibitor), reduces cell proliferation and induces apoptosis and senescence in vitro, resulting in the inhibition of tumor growth and progression *in vivo*. All these drugs and their effects or targets are summarized in Table 2.

## 5. Conclusions

The deregulation of gene expression by epigenetic alterations is recognized as an important feature of cancer, and knowledge of epigenetic regulation is a useful tool for the understanding of carcinogenesis, as well as for the development of anti-epigenetic drugs.

Targeting epigenetic modifications seems to be a novel approach contributing to precision medicine, although there are still limitations to be overcome before it reaches a clinical setting for treating cancer and other diseases.

The methylation of *HOX* genes or associated histones is recognized as a potential biomarker in several cancer types, facilitating predictions of disease outcome, and therefore, improving treatment decisions. The methylation of some *HOX* genes is also associated with therapy resistance, and thus, knowledge of its methylation profile may orientate the patients regarding treatment alternatives.

## 6. Future Perspectives

The flexibility of the epigenome has generated an appealing argument for the exploration of its reversion through pharmacological treatments and as a strategy to inhibit disease phenotypes, or even acting as radiosensitizers. In addition, epigenetic modifications may alter drug response in a very specific manner, leading to increased sensitivity or resistance to treatment. This led to an interest in developing “epidrugs”, some of which are already commercially available or in clinical trials [129,139]. These drugs have been successfully used in cancer treatment, frequently in combination with chemotherapy, and have been shown to cause cytotoxicity or inhibit resistance to anticancer drugs. However, their side effects are undesirable changes in epigenetic signatures which are poorly tolerated by patients. Therefore, the challenge is to uncover epidrugs with targeted effects or to establish the proper balance using combined therapeutic approaches.

Kits for gene-specific methylation detection in specific cancer types are increasing the feasibility of methylation analyses, including the use of nearly all body fluids, such as blood spots, bronchial aspirates, saliva or urine. Consequently, these analyses could be useful for early detection and/or progression screening in a non-invasive way. Tissue analysis is equally possible in almost all of them, including formalin-fixed paraffin-embedded (FFPE) tissue, but with one major difficulty: ensuring the cellular heterogeneity of the tumor. Another challenge is that in spite of the fact that different techniques are available for methylation analyses, the lack of standardized and reproducible protocols may impair the credibility of the resulting assays.

Despite the development of some chemical compounds to regulate DNA and histone methylation status [138,140], it is expected that drugs targeting specific epigenetic alterations, including those related to *HOX* genes, will appear in the near future. This is justified by the high level of importance that the alteration of *HOX* gene expression has in cancer predisposition and development, and by the fact that the generalized effect of some epigenetic drugs may lead to secondary malignancies.

## Figures and Tables

**Table 1:** Association between *HOX* gene hypermethylation and cellular processes involved in cancer progression.

Cancer Site	*HOX* Genes	Possible Roles	References
Bile duct	*HOXA1, HOXA2, HOXA5, HOXA11, HOXB4, HOXD9, HOXD13*	Biomarkers for the detection of cholangiocarcinoma in tissues or serum cell-free.	[31,32,33]
Bladder	*HOXA9*	Biomarker for the detection of bladder cancer and prediction response to cisplatin-based chemotherapy and survival.	[34,35]
	*HOXB2*	Biomarker to predict high-grade, noninvasive disease.	[36]
Blood (Leukemias/ Lymphomas)	*HOXA4*	Biomarker to predict resistance to imatinib mesylate.	[37]
	*HOXA4*, *HOXA5*	Biomarkers to predict progression to blast crisis.	[38]
	*HOXD8*	Targeted for therapeutic benefit in MCL (Mantle cell lymphoma).	[39]
Breast	*HOXA1*	Biomarker to distinguish different breast cancer states subgroups.	[24,40]
	*HOXA4*	Biomarker for early breast cancer detection.	[41]
	*HOXA5*	Biomarker specific to high-grade ductal carcinoma in situ detection and Triple-Negative breast cancer nonresponders to neoadjuvant chemotherapy.	[42,43]
	*HOXA9, HOXA10*	Biomarkers to predict survival.	[44]
	*HOXA10, HOXB13*	Biomarker to distinguish different breast cancer states subgroups with high expression of estrogen and progesterone receptors.	[24,40]
	*HOXA11*	Biomarker for unfavorable prognosis in breast cancer.	[45]
	*HOXB4*	Biomarker for metastatic breast cancer detected in circulating tumor cells.	[46]
	*HOXB13*	Biomarkers for the detection of breast cancer.	[47]
	*HOXC8*	Epigenetic downregulation interferes with stem cell transformation.	[48]
	*HOXC9*	Detected in breast cancer.	[49]
	*HOXC10*	Detected in endocrine-resistant breast cancer and associated with recurrence during aromatase inhibitor treatment.	[50]
	*HOXD1*	Biomarkers for the detection and prognosis of breast cancer.	[51]
	*HOXD11*	Detected in breast cancer.	[52]
	*HOXD13*	Biomarker for poor survival prognostic.	[53]
Cervix	*HOXA9*	Epigenetic downregulation relates to cell proliferation, migration and expression of epithelial-to-mesenchymal transition genes.	[54]
Colorectal	*HOXA2*	Epigenetic downregulation relates to lymphovascular invasion, perineural invasion, lymph node number.	[38]
	*HOXA5, HOXA6*	Epigenetic downregulation favors tumor progression.	[55]
	*HOXD3*	Identified in colorectal cancers.	[56]
	*HOXD10*	Epigenetic downregulation favors proliferation, migration, invasion and apoptosis.	[57]
Endometrial	*HOXA9, HOXD10*	Biomarker for detection of early onset of endometrial cancer.	[58]
Kidney	*HOXA5*	Epigenetic downregulation associated with high-grade clear cell renal cell carcinoma.	[59]
	*HOXA11*	Epigenetic downregulation associated with proliferation, colony formation, migration and invasion abilities in renal cell carcinoma.	[60]
	*HOXB13*	Epigenetic downregulation associated with reduced apoptosis and increased tumor grade and microvessel invasion in renal cell carcinoma.	[61]
Head and neck	*HOXA5*	Epigenetic downregulation favors invasion in nasopharyngeal cancer.	[62]
	*HOXB2*	Biomarker for lymph node metastasis in esophageal squamous cell carcinoma.	[63]
	*HOXA9*	Epigenetic downregulation associated with tumor progression and metastasization in head and neck squamous cell carcinoma and biomarker to distinguish oral cancer patients at low risk of neck metastasis.	[64,65]
	*HOXB4, HOXC4*	Biomarkers to predict survival of oral squamous cell carcinoma.	[66]
Liver	*HOXD10*	Epigenetic downregulation activates ERK signaling in hepatocellular carcinoma and causes vessel cancerous embolus and tumor cell differentiation.	[67]
	*HOXB4*	Epigenetic downregulation disruption of miR-10ª regulation hepatocellular carcinoma.	[68]
Lung	*HOXA1, HOXA11*	Biomarker involved in a molecular signature that helps to distinguish between atypical adenomatous hyperplasia, adenocarcinoma in situ and lung adenocarcinoma	[69]
	*HOXA2*	Biomarker involved in a molecular signature that helps to stratify lung squamous cell carcinoma into molecular subtypes with distinct prognoses.	[70]
	*HOXA2, HOXA10*	Biomarkers relevant for the prognosis of nonsmall cell lung cancer patients.	[71]
	*HOXA3*	Epigenetic downregulation in lung adenocarcinoma is associated with progression and poor prognosis.	[72]
	*HOXA5*	Epigenetic downregulation favors tumor-node-metastasis, tumor size, and lymph node metastasis in nonsmall cell lung cancer. It also favors invasion in lung adenocarcinomas.	[73,74]
	*HOXA5, HOXA10, HOXA4, HOXA7, HOXD13*	Identified in lung cancer.	[75]
	*HOXA7, HOXA9*	Epigenetic downregulation is associated with recurrence in nonsmall cell lung cancer. This alteration is part of a molecular signature relevant for detection and prognostic of primary nonsmall cell lung cancer using serum DNA.	[76,77]
	*HOXA11*	Epigenetic downregulation is associated with progression of nonsmall cell lung cancer. This alteration is part of a molecular signature involved in cell proliferation and migration in lung adenocarcinoma.	[78,79]
	*HOXB3, HOXB4*	Biomarkers in lung adenocarcinomas correlated with smoking history and chronic obstructive pulmonary disease.	[80]
	*HOXD3*	Biomarker for lung cancer.	[56]
	*HOXD8*	Epigenetic downregulation correlated with clinicopathological characteristics, cell migration and metastasization	[81]
	*HOXD10*	Biomarker to distinguish lung cancer, pulmonary fibrosis and chronic obstructive lung disease.	[82]
	*HOXD13*	Biomarker for lung adenocarcinoma.	[83]
Nervous System	*HOXA3, HOXA7, HOXA9, HOXA10*	Biomarkers to distinguish different glioma subgroups.	[84]
	*HOXA10*	Part of a stem cell related HOX-signature in glioblastoma.	[85]
	*HOXA11*	Epigenetic downregulation associated with treatment resistance and poor prognosis in glioblastoma.	[86]
	*HOXC4, HOXD8, HOXD13*	Biomarkers that distinguish long- and short-term glioblastoma survivors.	[87]
Ovaries	*HOXA9, HOXD11*	Epigenetic downregulation involved in DNA repair inactivation, cell cycle, apoptosis, cell adherence in ovarian cancer	[88]
	*HOXA9, HOXB5*	Identified in ovarian cancer and correlated with clinicopathological characteristics.	[89]
	*HOXA10, HOXA11*	Prognostic biomarker in ovarian cancer.	[90]
Prostate	*HOXA9*	Part of a molecular signature for prostate cancer clinical staging based on urine collection.	[91]
	*HOXD3*	Identified in prostate cancer and related to the development of high-grade tumors and recurrence	[92,93]
	*HOXD8*	Urine-based methylation biomarkers to predict prostate cancer progression.	[94]
Stomach	*HOXA1, HOXA10, HOXD10*	Biomarker for the diagnosis of gastric cancer.	[95]
	*HOXA10*	Interferes with miR-196b-5p-dependent proliferation and invasion of gastric cancer cells.	[30]
	*HOXA11*	Identified in gastric cancer and proposed to affect cell proliferation.	[96]
	*HOXB13*	Biomarker for gastric cancer involved in invasion depth, lymph node metastasis and tumor-node-metastasis stage.	[97]
	*HOXD1*	Biomarkers for predicting lymph node metastasis of stomach cancer.	[98]
	*HOXD10*	Epigenetic downregulation associated with gastric carcinogenesis.	[99]
Testis	*HOXA9*	Biomarker for testicular germ cell tumor subtyping.	[100]
Thyroid	*HOXA1*	Biomarker for the diagnosis of thyroid nodules.	[101]
	*HOXA7*	Biomarker for papillary thyroid cancer.	[102]
	*HOXB4*	Part of a molecular signature identifying biologically distinct thyroid cancer subtypes.	[103]
	*HOXD10*	Identified in papillary thyroid cancer with BRAFV600E mutation and associated with primary tumor invasion and age > 45.	[104]

**Table 2:** Drugs that may alter the methylation status of the DNA or histones associated with *HOX* gene regulation.

Drug Candidates	Target Molecule	Effects	References
Zebularine	DNMTs and cytidine deaminase	Alter pan-DNA methylation status	[129]
5-aza-deoxycytidine/5-azacytine	DNMTs	Alter pan-DNA methylation status	[128,129]
Vorinostat/Belinostat /panobinostat/Romidepsin/Chidamide	HDACs	Alter histones methylation status	[129]
GSK-J4	Histones	Inhibits the histone modulator KDM6B/JMJD3	[130]
AC1NOD4Q	Histones	Inhibits the HOX antisense intergenic RNA (HOTAIR)/EZH2 interaction	[131]
JQ1	HOX antisense intergenic RNA (HOTAIR)	Inhibits the HOX antisense intergenic RNA (HOTAIR) through the BET bromodomain inhibition	[132]
GSK-J1	Histones	Inhibits JMJD3 and UTX demethylases	[136]
GSK-J3	Histones	Targeting JMJD3 demethylase	[136]
